# Regional differences in foveal avascular zone morphology in cynomolgus macaques using a normative OCTA database

**DOI:** 10.1038/s41598-026-60198-2

**Published:** 2026-07-18

**Authors:** Claudia Bruè, Peter M. Maloca, Cathy Cukras, Philippe Valmaggia, Nora Denk

**Affiliations:** 1 Ophthalmic clinic, Macerata Hospital, Macerata, Italy; 2 Department of Ophthalmology, University Hospital Basel, Basel, 4031 Switzerland; 3 Department of Biomedical Engineering, University of Basel, Allschwil, 4123 Switzerland; 4 Moorfields Eye Hospital NHS Foundation Trust, London, EC1V 2PD UK; 5 Roche Pharma Research and Early Development Ophthalmology, Roche, US; 6 Pharma Research and Early Development (pRED), Pharmaceutical Sciences (PS), Roche Innovation Center Basel, Basel, Switzerland

**Keywords:** Foveal avascular zone, Vessel density, Cynomolgus macaques, Origin, Retina, Anatomy, Computational biology and bioinformatics, Diseases, Health care, Medical research

## Abstract

Cynomolgus macaques are widely used in preclinical ophthalmic research because of their close anatomical and physiological similarity to the human eye. However, the small cohort sizes typical of laboratory studies and the limited implementation of automated image analysis have restricted the availability of robust normative datasets. In particular, comprehensive reference values for foveal avascular zone (FAZ) measurements in laboratory cynomolgus monkeys remain scarce. To address this, an automated OCT/OCTA image-processing workflow was used to quantify FAZ morphology in healthy cynomolgus monkeys, including a deep learning-based OCT segmentation step followed by analysis of the corresponding OCTA data. The study also evaluated morphological variations as well as the influence of sex and geographical origin. The overall FAZ area measured 0.451 mm (range 0.132–0.820 mm^2^), the perimeter 2.770 mm (range 1.569–3.795), aspect-ratio 0.959 mm (range 0.622–1.388), circularity 0.725 (range 0.517–0.847), solidity 0.944 mm (range 0.816–0.991 mm). Moreover, geographical origin significantly affects FAZ area and aspect-ratio. Establishing a normative FAZ reference database is essential for the accurate interpretation of structural and vascular changes in translational ocular research and provides a quantitative framework to distinguish physiological variability from treatment-related effects, thereby supporting safety assessment and efficacy evaluation in preclinical drug development.

**Supplementary Information:**

The online version contains supplementary material available at 10.1038/s41598-026-60198-2.

## Introduction

The cynomolgus macaque (CM) has emerged as a widely utilized nonhuman primate model in preclinical ophthalmic drug development owing to its close genetic, anatomical, and physiological similarity to the human eye, particularly the presence of a well-defined fovea^1–5^. Significant attention has been devoted to investigating the fovea, the region responsible for the highest visual acuity^6^. Interestingly, both human and macaque foveae exhibit similar vascular structures, most notably the presence of a central foveolar avascular zone (FAZ)^6^. The fovea, which lacks overlying capillaries, along with FAZ parameters, has been identified as a valuable biomarker for the diagnosis and monitoring of retinal microvascular diseases such as diabetic retinopathy^7,8^. The fovea is the preferred site for neurodegenerative and circulatory diseases^9^. In part, this particular vulnerability may be caused by its vascular deprivation as the fovea is almost entirely dependent on appropriate blood perfusion across the choroid^10^.

Optical coherence tomography angiography (OCTA) is important for the analysis of the fovea and the FAZ because it enables high-resolution, depth-resolved, and non-invasive imaging of the retinal and choroidal microvasculature, including the superficial and deep capillary plexuses, which is crucial for the detection and characterization of vascular abnormalities in the fovea and throughout the retina^11,12^. In contrast to conventional dye-based angiography, OCTA allows visualization of the microvascular network without the risks associated with dye injection and provides three-dimensional assessment of blood flow, thereby facilitating the identification of ischemia, neovascularization, capillary dropout, and microaneurysms in diseases such as diabetic retinopathy and age-related macular degeneration^13–15^.

Accordingly, changes in the FAZ area may represent early biomarkers of retinal vascular pathology; however, existing reference values are predominantly based on cohorts of healthy human eyes and remain limited in scope. To overcome this constraint, the present study utilized OCTA to quantitatively assess FAZ parameters in a large cohort of healthy cynomolgus monkeys and to comprehensively characterize their morphological features, thereby establishing a robust normative dataset to facilitate future translational and preclinical investigations.

## Methods

### Animals and husbandry

This study utilized preexisting, retrospective data originally collected from routine investigations carried out during pharmaceutical product development. Consequently, no additional animal experimentation was conducted for this research. Specifically, OCTA scans from ocular safety studies involving treatment-naïve cynomolgus monkeys (Macaca fascicularis) of both sexes were analyzed retrospectively. The initial safety studies were reviewed and approved by the Institutional Animal Care and Use Committees (IACUC) of the respective organizations, including Charles River Laboratories Montreal (CR-MTL IACUC), Charles River Laboratories Reno (OLAW Assurance No. D16-00594), and Covance Laboratories Inc., Madison, WI, USA (OLAW Assurance #D16-00137 (A3218-01)). This study complies with the ARRIVE guidelines. All animals were handled and cared for in strict accordance with the guidelines established by the US National Research Council or the Canadian Council on Animal Care. These monkeys were specifically bred for laboratory use and sourced from certified suppliers located in two geographical regions: Mauritius and Asia. The animals were group-housed in stainless steel cages that meet European housing standards as outlined in Annex III of Directive 2010/63/EU.

## OCT image acquisition

Imaging was conducted under general anesthesia using ketamine (10 mg/kg intramuscularly) and dexmedetomidine (25 µg/kg intramuscularly) to reduce animal stress and maintain stable eye positioning. Just prior to OCTA imaging, a single intramuscular dose of midazolam (0.2 mg/kg) was given to ensure the eyes remained centrally aligned. Pupillary dilation was achieved through topical application of tropicamide before the imaging procedure. OCTA images were captured using a spectral-domain OCT system (Heidelberg Engineering, Heidelberg, Germany). For each OCTA scan, the corresponding (aligned) OCT volume was exported.

## Automated image processing

Initially, we employed a previously developed and validated deep learning-based algorithm healthy cynomolgus monkeys for the automated segmentation of the structural OCT scans into vitreous, retina, choroid, and sclera^16^. In short, and as previously demonstrated, compartmentalization of the OCT volumes was performed using a modified U-Net convolutional neural network (CNN) comprising 22 convolutional layers, five transposed convolutions, and five skip connections. The network was trained on a ground-truth dataset of 1,100 B-scans, independently annotated by three experienced retina specialists, and generated pixel-wise semantic segmentation maps for each B-scan, delineating four anatomical compartments: vitreous, retina, choroid, and sclera. The inner retinal boundary was defined as the internal limiting membrane, whereas the outer retinal boundary corresponded to the outer margin of the retinal pigment epithelium. Minor segmentation artifacts, consisting of small, misclassified pixel patches, were subsequently corrected on the assumption that the four largest connected regions within each segmentation map represented the four true anatomical compartments; any additional, smaller connected regions were therefore reassigned the label of the compartment immediately surrounding them. Because OCTA is generated from consecutive structural OCT B-scans acquired at the same anatomical location, the previously described compartmentalization of the structural OCT data can be directly transferred to the corresponding OCTA acquisition. Since both modalities share the same B-scan geometry and spatial coordinates, the boundaries of the defined compartments—vitreous, retina, choroid, and sclera—can be co-registered with the OCTA dataset without requiring additional segmentation. This enables the angiographic flow signal to be selectively extracted from within the retinal compartment. Consequently, OCTA data can be analyzed in an anatomically compartment-specific manner that remains fully consistent with the segmentation of the structural OCT data.

**Fig. 1:**
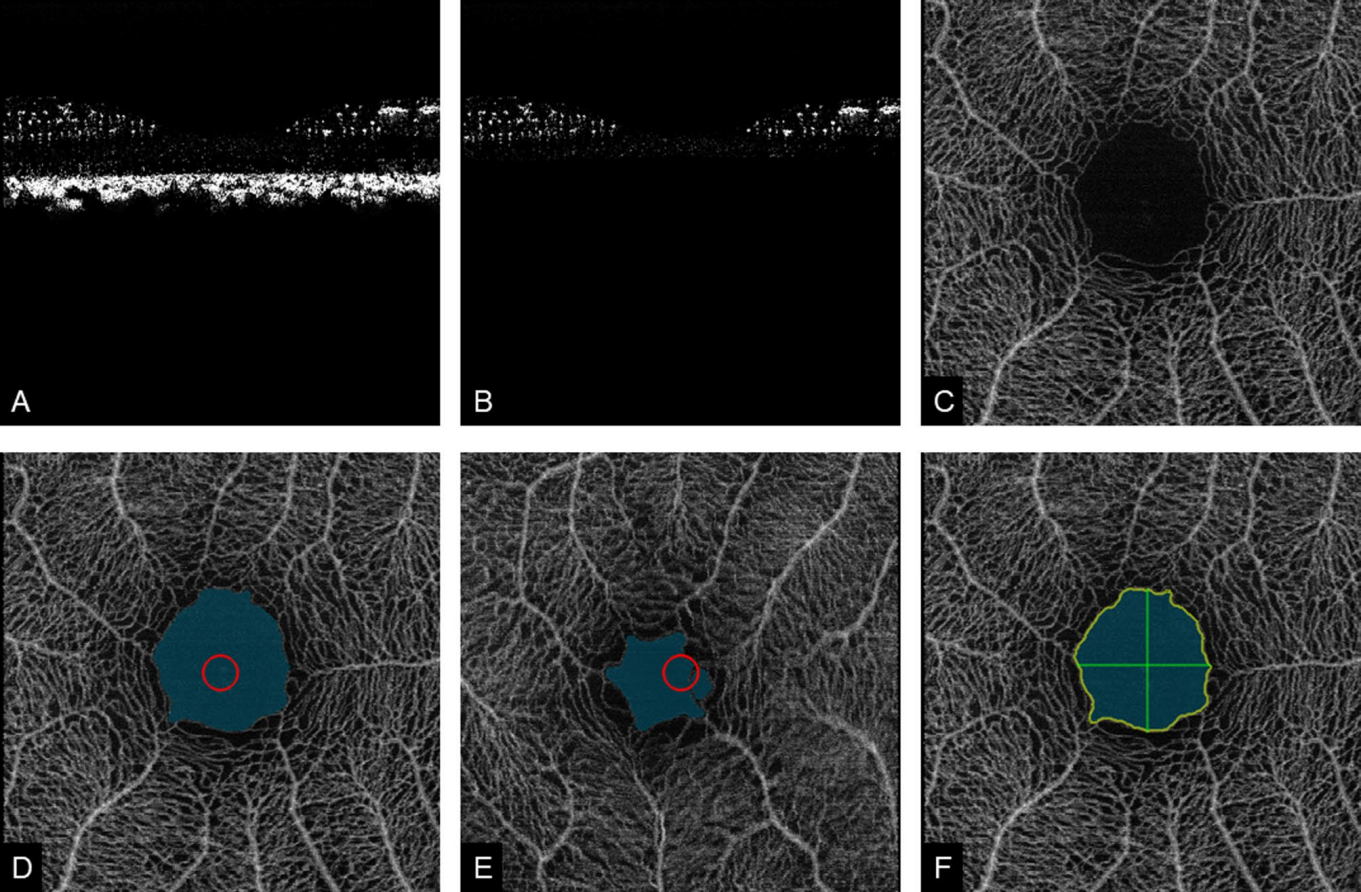
Overview of the algorithm for segmenting the foveolar avascular zone (FAZ). The initial OCTA scan (**A**) was masked using a compartment segmentation (vitreous, retina, choroid, sclera) from the corresponding registered OCT scan. This OCT segmentation was employed to mask all non-retinal voxels in the OCTA image (**B**). The masked OCTA image was subsequently used to generate the en-face image (**C**). The FAZ was segmented using the MorphACWE algorithm, with an initial search boundary defined as a red circle at the center (**D** and **E**). If the initial search boundary is not entirely within the FAZ, multiple segmented objects may arise. In such cases, only the largest object was retained. Finally, the geometric key coefficients are calculated (**F**).

Subsequently, these segmented OCT data were utilized to mask the vitreous, choroid, and sclera voxels in the corresponding registered OCTA images (see Fig. 1A). This masking process ensured that only the retinal voxels remained visible in the OCTA images (see Fig. 1B).

To ensure reliability of the image-processing workflow, the outputs of the automated steps were manually reviewed by two independent reviewers for quality and anatomical plausibility. In particular, the OCT compartment segmentations, retinal masking of the registered OCTA volumes, en-face projections, and final FAZ segmentations were visually inspected before quantitative analysis.

## En-face image generation

En-face images were generated from the masked OCTA images by summing the voxel intensities along the z-axis, as defined by the standard medical OCT coordinate system. The resulting two-dimensional array was then normalized to produce the final en-face image (Fig. 1C).

## FAZ segmentation

### Segmentation

The foveolar avascular zone in OCTA is defined as the central, capillary-free area at the level of the fovea, bounded by the surrounding perifoveal microvasculature. It is identified on en face OCTA images as a region devoid of detectable blood flow signal, typically corresponding anatomically to the site overlying the foveal depression where capillaries are physiologically absent to allow unobstructed light transmission to the photoreceptors. The FAZ boundary is most commonly delineated using automated or semi-automated segmentation algorithms provided by the respective OCTA device manufacturer, which trace the innermost margin of the surrounding capillary network rather than relying on a fixed axial depth or a single, predefined vascular plexus.

In the en-face image, the FAZ was segmented using a Morphological Active Contours without Edges (MorphACWE) algorithm [1]. MorphACWE is a region-based segmentation method that evolves the contour according to differences in image intensity between the inside and outside of the target region, rather than relying on strong edges. The initial search boundary was automatically placed at the center of the image as a circle with a radius of 20 pixels (red circle in Fig. 1D and E), and the algorithm was then executed for 240 iterations. The suitability of this FAZ segmentation approach was assessed qualitatively through review of representative segmentations by the study team, including an ophthalmology specialist, who confirmed that the segmentation quality was acceptable for the intended application.

### Post-processing

If the initial circular search boundary was not fully contained within the FAZ, contour evolution could occasionally converge to multiple disconnected candidate regions in the en-face image. In such cases, a post-processing step was applied in which only the largest connected component was retained as the final FAZ segmentation.

For the geometric analysis, the following coefficients of the segmented FAZ were calculated:

**Area** [mm2]: The area of the FAZ in the en-face image (blue area in Fig. 1F). **Perimeter** [mm]: The perimeter around the FAZ (yellow line in Fig. 1F). **Aspect-Ratio**: The ratio of the maximum horizontal diameter (horizontal green line in Fig. 1F) to the maximum vertical diameter (vertical green line in Fig. 1F). **Circularity**: A measure of the resemblance of the FAZ to a circle, calculated as $\:4\pi\:\:*\:{Area}_{FAZ}\:/\:{Perimeter}^{2}$. A value of 1.0 indicates a perfect circle, while lower values indicate increasing deviation from circularity. **Solidity**: A measure of the concavity or texture of the FAZ. Calculated as: $\:{Area}_{FAZ}\:/\:{Area}_{convex}$, where $\:{Area}_{convex}$ is the area of the convex hull of the FAZ. The highest value of 1.0 indicates a completely convex FAZ with no concavities. Lower values indicate more irregular, or “holey” FAZ shapes with significant concavities (the convex hull area is much larger than the actual area of the object). Circularity and solidity are both dimensionless indices ranging from 0 to 1, each capturing a distinct geometric property of the FAZ outline. Circularity quantifies the overall resemblance of the FAZ to a perfect circle, penalising both elongation and border irregularity; a value of 1.0 denotes a perfect circle, while lower values reflect increasingly non-circular shapes. Solidity specifically measures the degree of concave indentation or lobulation of the FAZ border, independently of overall shape elongation; a value of 1.0 indicates a fully convex outline with no concavities, while lower values indicate progressive border notching. Importantly, the two indices are complementary: a smooth but elongated FAZ may retain near-unity solidity while showing reduced circularity, whereas a lobulated FAZ with localised concavities will show reduced solidity regardless of its overall roundness.

This distinction matters clinically, as early neovascular or ischaemic changes often produce localised notching before the overall shape becomes grossly non-circular.

All automated image processing steps were executed in Python v3.8. For implementing the MorphACWE algorithm, the blob removal process, and calculating the geometric key coefficients, scikit-image v0.17.2(1) was utilized.

### Data analysis

#### Summary statistics and visualization

Summary statistics, including the mean, standard deviation, and quartiles, were calculated for each of the geometric coefficients. Boxplots were employed to visualize data distribution and facilitate comparisons between different groups, such as male and female, and individuals from Asia and Mauritius.

### Statistical analysis

To investigate the observed variability in the data and to investigate differences with respect to sex and origin, the following statistical analyses were performed:

### Correlation analysis

A Pearson correlation analysis was performed as an exploratory step to assess dependencies and potential redundancy among the geometric FAZ descriptors area, perimeter, aspect-ratio, circularity, and solidity prior to inferential testing. Because area and perimeter were highly correlated, perimeter was not included in the subsequent Analysis of Variance (ANOVA). Similarly, because circularity and solidity were highly correlated, solidity was not included in the subsequent ANOVA.

### Analysis of Variance (ANOVA)

A separate ANOVA was performed for each of the dependent variables: area, aspect-ratio, and circularity. The independent variables were sex, geographic origin, and ocular laterality (left or right eye). Type II sum of squares was used for ANOVA since it is more sensitive to the main effects. To adjust for the multiple testing problem, we performed Bonferroni correction by dividing significance levels by the number of tests, which in our case was twelve (Three ANOVA with four *p*-values each). Thus, the significance levels 0.001, 0.01, and 0.05 became 0.001/12, 0.01/12, and 0.05/12. ANOVA assumptions were checked with quantile-quantile plots.

All summary statistics, data visualizations and ANOVA were performed in Python v3.8. Statistical tests were performed with the Python package statsmodels v0.14.5 and the visualizations were done with the Python packages matplotlib v3.10.7 and seaborn v0.13.2.

## Results

**Fig. 2:**
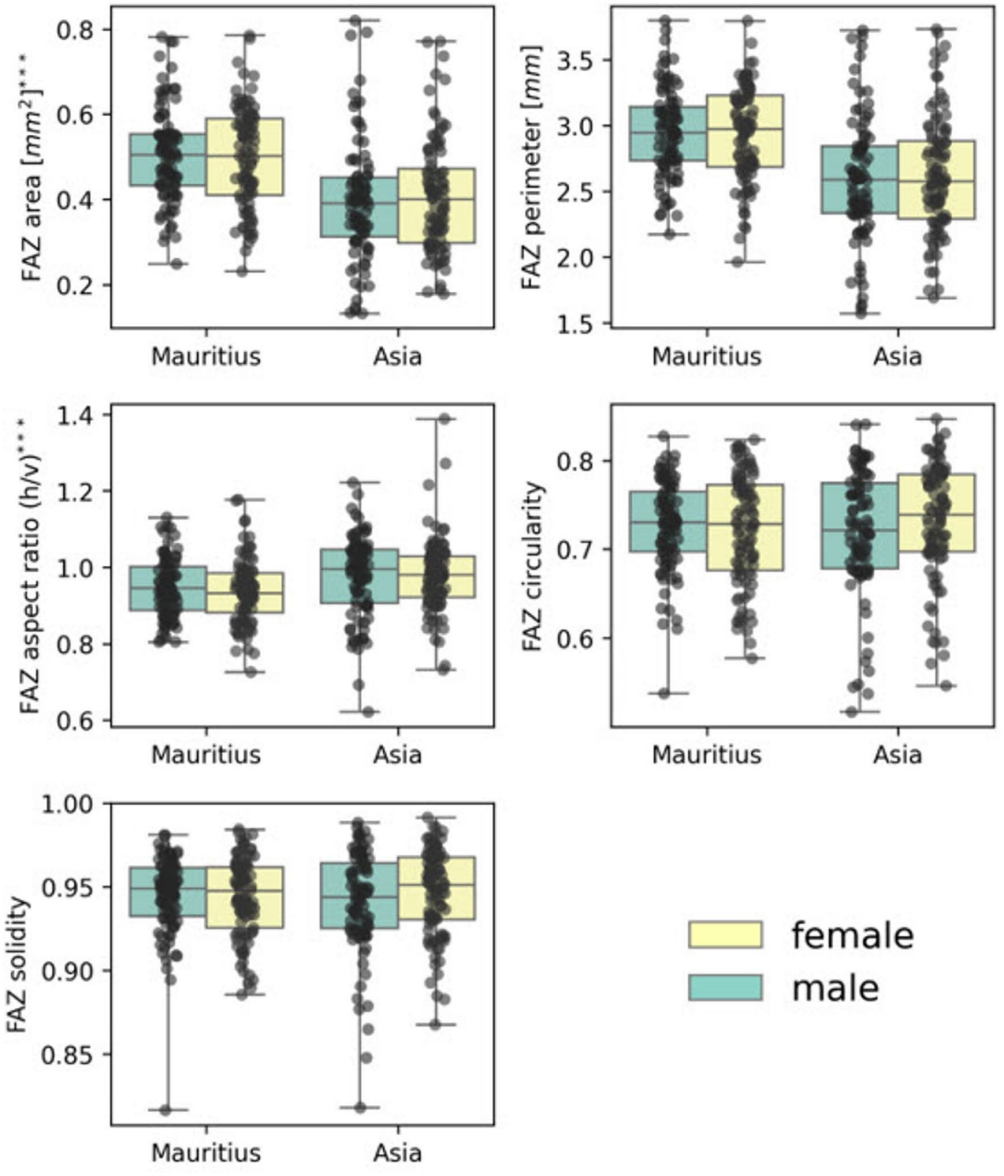
Distributions of five computed FAZ coefficients: area (top left), perimeter (top right), aspect ratio (horizontal/vertical; center left), circularity (center right), and solidity (bottom left). Boxplots are overlaid with univariate scatter (strip) plots showing individual observations. Female and male cohorts are shown separately. Three asterisks indicate statistically significant ANOVA results for the dependent variable origin (Mauritius vs. Asia), for area and aspect ratio as independent variables, using a Bonferroni-corrected threshold of *p* < 0.001/12.

In total, 368 OCTA measurements were provided from a total of 187 animals (189 eyes (51.36%) from 96 Female animals and 179 eyes (48.64%) from 91 male animals). Overall, 183 and 185 eyes were left (49.72%) and right (50.27%), respectively. Mauritian and Asian Monkeys were 93 and 94 individuals, respectively, who contributed 184 eyes each (50%). Sixteen eye OCTAs were excluded because image quality and/or segmentation quality was insufficient for reliable processing and FAZ quantification. The distribution of the five computed geometric FAZ coefficients area, perimeter, aspect-ratio, circularity, and solidity is shown in Fig. 2 and summarized in Supplementary Table 1. The effects of the four independent variables — sex, geographic origin, ocular laterality, and individual subject — on each outcome measure are illustrated as stars in Fig. 2, with statistically significant results marked by asterisks. The dependent variable was area in the first ANOVA, aspect ratio in the second, and circularity in the third. Significance codes denote levels of statistical significance: ***p < 0.001/12, **p < 0.01/12, *p < 0.05/12, with a Bonferroni correction applied by dividing the initial significance thresholds by 12.

**Fig. 3:**
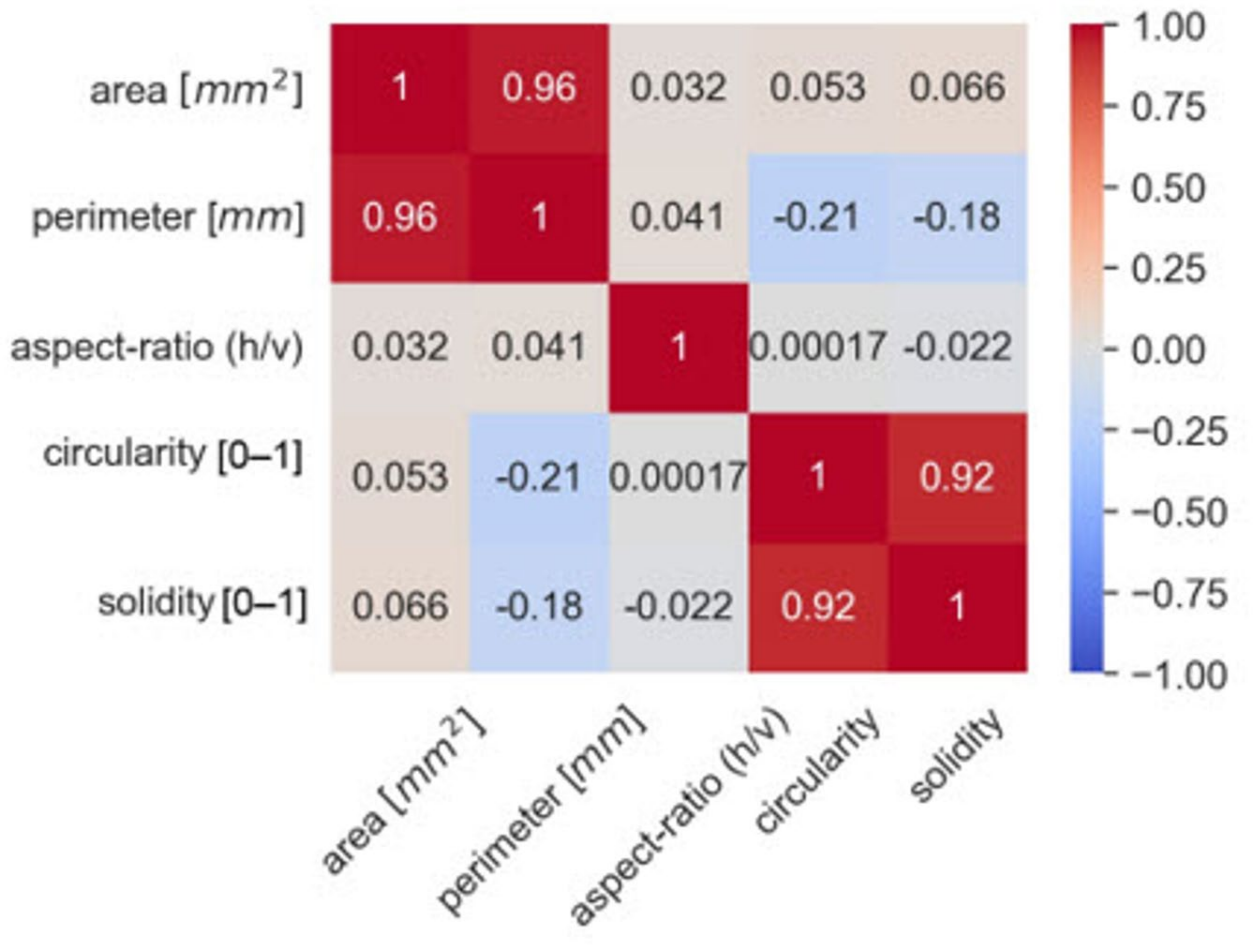
Pearson correlation analysis among the five geometric FAZ coefficients: area, perimeter, aspect-ratio, circularity, and solidity. As expected for related geometric descriptors, a very strong correlation was observed between area and perimeter (*r* = 0.96), as well as between circularity and solidity (*r* = 0.92). Circularity is a dimensionless index; 1.0 = perfect circle. A solidity value of 1.0 indicates a completely convex FAZ with no concavities.

The correlation analysis revealed a very strong Pearson correlation between the FAZ coefficients area and perimeter (*r* = 0.96), as well as between circularity and solidity (*r* = 0.92). The strong area–perimeter association was expected, as both metrics describe related geometric properties of the same structure. The correlation analysis was therefore used primarily to identify redundant descriptors prior to ANOVA, and perimeter and solidity were excluded from the subsequent models. Other observed correlations were predominantly minor (Fig. 3). The ANOVA identified three significant effects: the influence of origin on area, the influence of origin on aspect-ratio, and the influence of individual on area (Supplementary Table 2, Fig. 2).

## Discussion

The foveal avascular zone is a capillary-free region in the central macula essential for normal visual function. Previous studies using fundus fluorescein angiography in healthy humans reported mean FAZ areas ranging from 0.27 to 0.42 mm²^4^. However, fluorescein angiography is invasive and time-consuming and may be contraindicated in patients with renal impairment or fluorescein allergy. By contrast, in healthy humans the FAZ area is typically assessed using OCTA and averages approximately 0.3 mm² in the superficial capillary plexus, with reported ranges between 0.23 and 0.32 mm² depending on age, sex, and measurement technique^17–20^. Notably, the present study demonstrated that, relative to humans, cynomolgus macaques exhibit modestly larger OCTA-derived FAZ dimensions, with a mean area of 0.450 mm² (range 0.132–0.820 mm) and a mean perimeter of 2.770 mm (range 1.569–3.795 mm). It’s important to note that measurements can vary between different OCTA instruments, so values should be interpreted using device-specific normative data^21^. However, given the differences in OCTA devices, segmentation algorithms, scan protocols, and sample characteristics between the present study and previously published human cohorts, this comparison should be interpreted with caution, and the observed discrepancy cannot be unequivocally attributed to a true interspecies difference in FAZ dimensions. Direct, prospective comparisons using standardized acquisition and segmentation protocols across species would be required to confirm whether this apparent difference reflects a genuine biological distinction rather than methodological variability.

The establishment of a normative FAZ reference database is of substantial importance in both preclinical and translational research. Robust baseline values enable the differentiation between physiological variability and disease- or treatment-related alterations in retinal microvascular architecture. Such reference data may serve as safety parameters in drug development, particularly for compounds with potential vascular effects, and facilitate the early detection of ischemic or perfusion-related changes. Moreover, standardized FAZ metrics provide a quantitative framework for evaluating therapeutic efficacy or adverse vascular responses, thereby strengthening the interpretability and regulatory relevance of preclinical ocular studies.

In addition, this study demonstrated that cynomolgus monkeys of Mauritian origin exhibit a significantly larger FAZ area compared to animals of Asian origin, indicating that geographic ancestry represents a relevant biological determinant of retinal microvascular morphology. Although regional differences in foveal morphology have been described in earlier studies, the current investigation underscores and refines these observations by revealing corresponding alterations at the retinal microvascular level^16^. This finding is of particular importance for future preclinical investigations, as it underscores the need to account for population background when establishing normative datasets, designing experimental cohorts, and interpreting vascular endpoints. Failure to consider origin-specific differences may confound inter-study comparisons and obscure subtle treatment-related effects, especially in studies evaluating ischemia-modulating or vasoactive compounds. Accordingly, the normative FAZ values reported here should be interpreted primarily as a reference framework for studies in cynomolgus macaques performed under comparable imaging and analysis conditions, rather than as universally transferable thresholds.

Furthermore, the aspect ratio is significantly influenced by the origin of the monkeys. Monkeys of Asian descent exhibit an aspect ratio close to 1.0, whereas those from Mauritius display a ratio less than 1.0. This suggests that the FAZ in Mauritian monkeys extends more in the inferior-superior direction compared to the nasal-temporal direction.

These findings have important implications for the use of cynomolgus monkeys in preclinical drug research, particularly in studies related to ocular health and diseases. The distinct anatomical differences in FAZ morphology between monkeys from Mauritius and Asia may influence the baseline ocular parameters and how these animals respond to ophthalmic drugs. Understanding these variations is crucial for interpreting preclinical efficacy and safety data accurately and could aid in better translation of results from non-human primates to humans.

This study has several limitations. First, reproducibility and follow-up analysis could not be performed due to the structure of the available dataset. However, assessment of repeatability was not the primary objective of this study. Previous investigations have already demonstrated excellent repeatability of OCTA-based FAZ measurements in humans^22–24^. It is therefore reasonable to assume that this technology is similarly transferable and reproducible in animal models.

A further limitation was that only a single OCT device was used and no inter-device comparison was performed. Therefore, it should be emphasized that the reported values are specific to the device employed in this study and may not be directly transferable across different OCT platforms. However, the medical literature demonstrates that FAZ area measurements are highly consistent across devices, with intraclass correlation coefficients (ICC) typically above 0.95, making FAZ a reliable metric for multicenter studies and clinical follow-up^24–27^. An additional limitation is the absence of longitudinal data, and the timing of the measurements may have introduced diurnal fluctuations that could have influenced the results^28,29^. The medical literature demonstrates a clear relationship between OCTA metrics, axial length, and ocular magnification^30,31^. Axial length significantly affects quantitative OCTA measurements, including vessel density, perfusion density, and foveal avascular zone (FAZ) area. Longer axial length is associated with lower macular and optic nerve microvascular parameters, such as superficial and deep vessel density, and radial peripapillary capillary density, as well as a smaller FAZ area in healthy individuals^32,33^. Unfortunately, axial eye length was not available; therefore, it was not possible in this study to correlate vessel density with axial length. Human imaging is usually performed in awake subjects. In contrast, monkeys are typically imaged under general anesthesia, which may alter systemic blood pressure, ocular perfusion pressure, and retinal blood flow, potentially affecting vessel density measurements. Species-specific differences in vascular autoregulation and metabolic demand may lead to inherent differences in capillary density and FAZ morphology independent of disease. Most OCTA platforms are developed and calibrated based on human retinal architecture. Consequently, automated segmentation of retinal compartments in macaques may not perfectly correspond to the actual retinal layer architecture in macaques and could introduce systematic measurement bias.

## Conclusion

In conclusion, this study provides a comprehensive analysis of the FAZ in a large cohort of 187 cynomolgus monkeys, highlighting significant geographical differences in FAZ morphology between populations from Mauritius and Asia. Our findings reveal that Mauritian monkeys exhibit a larger FAZ area and a distinct aspect ratio compared to their Asian counterparts, suggesting variations in ocular anatomy that could impact the interpretation of preclinical drug research. Given the role of the FAZ in retinal health and visual acuity, these anatomical insights may enhance the translational value of cynomolgus monkeys as models for human ocular diseases, particularly in OCTA-based studies performed under comparable acquisition and analysis conditions. Further research is warranted to explore the genetic and environmental factors underlying these differences, which may inform the development of more tailored and effective therapeutic strategies.

## Supplementary Information

Below is the link to the electronic supplementary material.

Supplementary Material 1

Supplementary Material 2

## Data Availability

The datasets generated and/or analyzed during the current study are not publicly available due to institutional and regulatory restrictions but are available from the corresponding author upon reasonable request and with permission of the relevant ethics committee.

## References

[1] Satkoski Trask, J., George, D., Houghton, P., Kanthaswamy, S. & Smith, D. G. Population and landscape genetics of an introduced species (M. fascicularis) on the island of Mauritius. *PLoS One*. **8**, e53001. 10.1371/journal.pone.0053001 (2013). 23341917 10.1371/journal.pone.0053001 PMC3544817

[2] Tobias, P. et al. Safety and Toxicology of Ocular Gene Therapy with Recombinant AAV Vector rAAV.hCNGA3 in Nonhuman Primates. *Hum. Gene Ther. Clin. Dev.* **30**, 50–56. 10.1089/humc.2018.188 (2019). 30864850 10.1089/humc.2018.188

[3] Krebs, W. & Krebs, I. P. Quantitative morphology of the central fovea in the primate retina. *Am. J. Anat.* **184**, 225–236. 10.1002/aja.1001840306 (1989). 2750678 10.1002/aja.1001840306

[4] Wu, L. Z., Huang, Z. S., Wu, D. Z. & Chan, E. Characteristics of the capillary-free zone in the normal human macula. *Jpn J. Ophthalmol.* **29**, 406–411 (1985). 3831489

[5] Adamson, P. et al. Single ocular injection of a sustained-release anti-VEGF delivers 6months pharmacokinetics and efficacy in a primate laser CNV model. *J. Control Release*. **244**, 1–13. 10.1016/j.jconrel.2016.10.026 (2016). 27810558 10.1016/j.jconrel.2016.10.026 PMC5494198

[6] Anger, E. M. et al. Ultrahigh resolution optical coherence tomography of the monkey fovea. Identification of retinal sublayers by correlation with semithin histology sections. *Exp. Eye Res.* **78**, 1117–1125. 10.1016/j.exer.2004.01.011 (2004). 15109918 10.1016/j.exer.2004.01.011

[7] Conrath, J., Giorgi, R., Ridings, B. & Raccah, D. Metabolic factors and the foveal avascular zone of the retina in diabetes mellitus. *Diabetes Metab.* **31**, 465–470. 10.1016/s1262-3636(07)70217-3 (2005). 16357790 10.1016/s1262-3636(07)70217-3

[8] John, D., Kuriakose, T., Devasahayam, S. & Braganza, A. Dimensions of the foveal avascular zone using the Heidelberg retinal angiogram-2 in normal eyes. *Indian J. Ophthalmol.* **59**, 9–11. 10.4103/0301-4738.73706 (2011). 21157065 10.4103/0301-4738.73706 PMC3032258

[9] Bek, T. Regional morphology and pathophysiology of retinal vascular disease. *Prog Retin Eye Res.* **36**, 247–259. 10.1016/j.preteyeres.2013.07.002 (2013). 23892140 10.1016/j.preteyeres.2013.07.002

[10] Spaide, R. F. CHOROIDAL BLOOD FLOW: Review and Potential Explanation for the Choroidal Venous Anatomy Including the Vortex Vein System. *Retina* **40**, 1851–1864. 10.1097/iae.0000000000002931 (2020). 32740492 10.1097/IAE.0000000000002931

[11] Kashani, A. H. et al. Optical coherence tomography angiography: A comprehensive review of current methods and clinical applications. *Prog Retin Eye Res.* **60**, 66–100. 10.1016/j.preteyeres.2017.07.002 (2017). 28760677 10.1016/j.preteyeres.2017.07.002 PMC5600872

[12] Spaide, R. F., Fujimoto, J. G., Waheed, N. K., Sadda, S. R. & Staurenghi, G. Optical coherence tomography angiography. *Prog Retin Eye Res.* **64**, 1–55. 10.1016/j.preteyeres.2017.11.003 (2018). 29229445 10.1016/j.preteyeres.2017.11.003 PMC6404988

[13] Javed, A. et al. Optical coherence tomography angiography: a review of the current literature. *J. Int. Med. Res.* **51**10.1177/03000605231187933 (2023). 10.1177/03000605231187933 PMC10387790 37498178

[14] Sambhav, K., Grover, S. & Chalam, K. V. The application of optical coherence tomography angiography in retinal diseases. *Surv. Ophthalmol.* **62**, 838–866. 10.1016/j.survophthal.2017.05.006 (2017). 28579550 10.1016/j.survophthal.2017.05.006

[15] Faatz, H. & Lommatzsch, A. Overview of the Use of Optical Coherence Tomography Angiography in Neovascular Age-Related Macular Degeneration. *J. Clin. Med.* **13**10.3390/jcm13175042 (2024). 10.3390/jcm13175042 PMC11396513 39274255

[16] Maloca, P. M. et al. Uncovering of intraspecies macular heterogeneity in cynomolgus monkeys using hybrid machine learning optical coherence tomography image segmentation. *Sci. Rep.* **11**, 20647. 10.1038/s41598-021-99704-z (2021). 34667265 10.1038/s41598-021-99704-z PMC8526684

[17] Fujiwara, A. et al. Factors affecting foveal avascular zone in healthy eyes: An examination using swept-source optical coherence tomography angiography. *PLoS One*. **12**, e0188572. 10.1371/journal.pone.0188572 (2017). 29176837 10.1371/journal.pone.0188572 PMC5703551

[18] Heidarzadeh, H. R. et al. The central retina vessel density and foveal avascular zone values of 792 healthy adults using optical coherence tomography angiography. *Eye (Lond)*. **38**, 3434–3442. 10.1038/s41433-024-03320-w (2024). 39289520 10.1038/s41433-024-03320-w PMC11621524

[19] Zhou, L. et al. Quantitative assessment and determinants of foveal avascular zone in healthy volunteers. *J. Int. Med. Res.* **49**, 3000605211014994. 10.1177/03000605211014994 (2021). 33990149 10.1177/03000605211014994 PMC8127766

[20] Scanlon, G., O’Shea, S., Amarandei, G. & Butler, J. S. O’Dwyer, V. Investigation of factors that may affect the foveal avascular zone: An optical coherence tomography angiography study. *Optom. Vis. Sci.* **101**, 276–283. 10.1097/opx.0000000000002129 (2024). 38857040 10.1097/OPX.0000000000002129

[21] Shiihara, H. et al. Reproducibility and differences in area of foveal avascular zone measured by three different optical coherence tomographic angiography instruments. *Sci. Rep.* **7**, 9853. 10.1038/s41598-017-09255-5 (2017). 28851930 10.1038/s41598-017-09255-5 PMC5575252

[22] Lei, J. et al. Repeatability and Reproducibility of Superficial Macular Retinal Vessel Density Measurements Using Optical Coherence Tomography Angiography En Face Images. *JAMA Ophthalmol.* **135**, 1092–1098. 10.1001/jamaophthalmol.2017.3431 (2017). 28910435 10.1001/jamaophthalmol.2017.3431 PMC5710485

[23] You, Q. et al. *Retina* **37**, 1475–1482, doi:10.1097/iae.0000000000001407 (2017). 27930458 10.1097/IAE.0000000000001407 PMC5902313

[24] Lei, J., Pei, C., Wen, C. & Abdelfattah, N. S. Repeatability and Reproducibility of Quantification of Superficial Peri-papillary Capillaries by four Different Optical Coherence Tomography Angiography Devices. *Sci. Rep.* **8**, 17866. 10.1038/s41598-018-36279-2 (2018). 30552361 10.1038/s41598-018-36279-2 PMC6294753

[25] Lu, Y. et al. A quantitative comparison of four optical coherence tomography angiography devices in healthy eyes. *Graefes Arch. Clin. Exp. Ophthalmol.* **259**, 1493–1501. 10.1007/s00417-020-04945-9 (2021). 32975683 10.1007/s00417-020-04945-9

[26] Lu, Y. et al. Quantitative Comparison Of Microvascular Metrics On Three Optical Coherence Tomography Angiography Devices In Chorioretinal Disease. *Clin. Ophthalmol.* **13**, 2063–2069. 10.2147/opth.S215322 (2019). 31749603 10.2147/OPTH.S215322 PMC6816077

[27] Munk, M. R. et al. OCT-angiography: A qualitative and quantitative comparison of 4 OCT-A devices. *PLoS One*. **12**, e0177059. 10.1371/journal.pone.0177059 (2017). 28489918 10.1371/journal.pone.0177059 PMC5425250

[28] Lal, B. et al. Changes in Retinal Optical Coherence Tomography Angiography Indexes Over 24 Hours. *Invest. Ophthalmol. Vis. Sci.* **63**, 25. 10.1167/iovs.63.3.25 (2022). 35348589 10.1167/iovs.63.3.25 PMC8976927

[29] Lal, B., Alonso-Caneiro, D., Read, S. A. & Carkeet, A. Diurnal changes in choroidal optical coherence tomography angiography indices over 24 hours in healthy young adults. *Sci. Rep.* **13**, 3551. 10.1038/s41598-023-30433-1 (2023). 36864086 10.1038/s41598-023-30433-1 PMC9981752

[30] Youssef, M. M., Sadek, S. H. & Hatata, R. M. Macular and Optic Nerve Microvascular Alteration in Relation to Axial Length, by Optical Coherence Tomography Angiography (OCTA). *Clin. Ophthalmol.* **16**, 885–892. 10.2147/opth.S354235 (2022). 35345824 10.2147/OPTH.S354235 PMC8957341

[31] Sampson, D. M. et al. Axial Length Variation Impacts on Superficial Retinal Vessel Density and Foveal Avascular Zone Area Measurements Using Optical Coherence Tomography Angiography. *Invest. Ophthalmol. Vis. Sci.* **58**, 3065–3072. 10.1167/iovs.17-21551 (2017). 28622398 10.1167/iovs.17-21551

[32] Ito, Y. et al. Effects of age, sex, and axial length on macular structure and perfusion in normal eyes. *Med. (Baltim).* **105**, e49020. 10.1097/md.0000000000049020 (2026). 10.1097/MD.0000000000049020 PMC13225564 42216353

[33] Wang, T. et al. Evaluation of retinal vascular density and related factors in youth myopia without maculopathy using OCTA. *Sci. Rep.* **11**, 15361. 10.1038/s41598-021-94909-8 (2021). 34321564 10.1038/s41598-021-94909-8 PMC8319333

