# Supplementary material for: Regional differences in foveal avascular zone morphology in cynomolgus macaques using a normative OCTA database
Source: Sci Rep. 2026 Jul 18;16:22967. doi: 10.1038/s41598-026-60198-2 (PMC13392401; doi:10.1038/s41598-026-60198-2)
Supplement: Supplementary file 1 — Supplementary Material 1 [file 41598_2026_60198_MOESM1_ESM.docx]

|  |  | female Asia | female Mauritius | male Asia | male Mauritius | overall |
| --- | --- | --- | --- | --- | --- | --- |
| count |  | 96 | 93 | 88 | 91 | 368 |
| area [mm2] | mean | 0.406 | 0.499 | 0.394 | 0.504 | 0.451 |
|  | std | 0.131 | 0.119 | 0.141 | 0.11 | 0.135 |
|  | min | 0.178 | 0.231 | 0.132 | 0.248 | 0.132 |
|  | 25% | 0.298 | 0.41 | 0.313 | 0.433 | 0.352 |
|  | 50% | 0.4 | 0.501 | 0.39 | 0.504 | 0.440 |
|  | 75% | 0.472 | 0.59 | 0.452 | 0.553 | 0.547 |
|  | max | 0.771 | 0.785 | 0.82 | 0.781 | 0.820 |
| perimeter [mm] | mean | 2.62 | 2.93 | 2.59 | 2.94 | 2.770 |
|  | std | 0.448 | 0.362 | 0.473 | 0.336 | 0.440 |
|  | min | 1.69 | 1.96 | 1.57 | 2.17 | 1.569 |
|  | 25% | 2.29 | 2.68 | 2.33 | 2.73 | 2.459 |
|  | 50% | 2.57 | 2.97 | 2.59 | 2.94 | 2.767 |
|  | 75% | 2.88 | 3.23 | 2.84 | 3.14 | 3.074 |
|  | max | 3.73 | 3.79 | 3.72 | 3.79 | 3.795 |
| aspect-ratio (h/v) | mean | 0.978 | 0.937 | 0.976 | 0.946 | 0.959 |
|  | std | 0.0954 | 0.0845 | 0.108 | 0.076 | 0.093 |
|  | min | 0.731 | 0.726 | 0.622 | 0.805 | 0.622 |
|  | 25% | 0.921 | 0.882 | 0.906 | 0.887 | 0.899 |
|  | 50% | 0.979 | 0.932 | 0.995 | 0.945 | 0.961 |
|  | 75% | 1.03 | 0.985 | 1.05 | 1 | 1.020 |
|  | max | 1.39 | 1.18 | 1.22 | 1.13 | 1.388 |
| circularity | mean | 0.731 | 0.722 | 0.719 | 0.726 | 0.725 |
|  | std | 0.0651 | 0.0621 | 0.0707 | 0.0512 | 0.063 |
|  | min | 0.546 | 0.577 | 0.517 | 0.538 | 0.517 |
|  | 25% | 0.697 | 0.676 | 0.678 | 0.697 | 0.689 |
|  | 50% | 0.738 | 0.728 | 0.721 | 0.73 | 0.729 |
|  | 75% | 0.784 | 0.773 | 0.775 | 0.765 | 0.775 |
|  | max | 0.847 | 0.823 | 0.84 | 0.827 | 0.847 |
| solidity | mean | 0.947 | 0.944 | 0.941 | 0.945 | 0.944 |
|  | std | 0.0261 | 0.0242 | 0.0312 | 0.0237 | 0.026 |
|  | min | 0.868 | 0.885 | 0.818 | 0.816 | 0.816 |
|  | 25% | 0.931 | 0.925 | 0.925 | 0.932 | 0.929 |
|  | 50% | 0.951 | 0.947 | 0.944 | 0.949 | 0.948 |
|  | 75% | 0.968 | 0.962 | 0.964 | 0.961 | 0.964 |
|  | max | 0.991 | 0.984 | 0.988 | 0.981 | 0.991 |

Fig. Supplementary 1. Summary statistics of the five coefficients area, perimeter, aspect-ratio, circularity, and solidity, calculated separately for female Asia eyes, female Mauritius eyes, male Asian eyes, male Mauritius eyes, and overall.
