# Supplementary material for: Regional differences in foveal avascular zone morphology in cynomolgus macaques using a normative OCTA database
Source: Sci Rep. 2026 Jul 18;16:22967. doi: 10.1038/s41598-026-60198-2 (PMC13392401; doi:10.1038/s41598-026-60198-2)
Supplement: Supplementary file 2 — Supplementary Material 2 [file 41598_2026_60198_MOESM2_ESM.docx]

| FAZ area [mm^2] | variable | sum_sq | df | F | PR(>F) |
| --- | --- | --- | --- | --- | --- |
|  | sex | 0.00722 | 1 | 0.479 | 0.489 |
|  | origin | 0.558 | 1 | 37 | 2.95E-09 |
|  | ocular laterality | 0.00132 | 1 | 0.0876 | 0.767 |
|  | individual | 0.301 | 1 | 20 | 0.0000103 |
|  | residual | 5.47 | 363 | NaN | NaN |
|  |  |  |  |  |  |
| FAZ aspect ratio (h/v) | variable | sum_sq | df | F | PR(>F) |
|  | sex | 0.00024 | 1 | 0.0288 | 0.865 |
|  | origin | 0.142 | 1 | 17.1 | 0.0000447 |
|  | ocular laterality | 0.00104 | 1 | 0.124 | 0.725 |
|  | individual | 0.0252 | 1 | 3.02 | 0.083 |
|  | residual | 3.03 | 363 | NaN | NaN |
|  |  |  |  |  |  |
| FAZ circularity | variable | sum_sq | df | F | PR(>F) |
|  | sex | 0.00074 | 1 | 0.19 | 0.663 |
|  | origin | 0.00109 | 1 | 0.28 | 0.597 |
|  | ocular laterality | 0.000113 | 1 | 0.0291 | 0.865 |
|  | individual | 0.0193 | 1 | 4.96 | 0.0265 |
|  | residual | 1.41 | 363 | NaN | NaN |

Fig. Supplementary 2. Results of three ANOVA. All analyses use independent variables sex, origin, ocular laterality, and individual. One ANOVA was performed for each of the independent variables: FAZ area [mm^2], FAZ aspect ratio (h/v), and FAZ circularity. Column definitions: sum_sq = sum of squares attributable to each factor; df = degrees of freedom; F = F-statistic; PR(>F) = p-value for the F-test.
